# Multiple-pinhole collimators improve intra- and between-rater agreement and the certainty of the visual interpretation in dopamine transporter SPECT

**DOI:** 10.1186/s13550-022-00923-w

**Published:** 2022-08-17

**Authors:** Franziska Mathies, Ivayla Apostolova, Lena Dierck, Janin Jacobi, Katja Kuen, Markus Sauer, Michael Schenk, Susanne Klutmann, Attila Forgács, Ralph Buchert

**Affiliations:** 1 Department of Diagnostic and Interventional Radiology and Nuclear Medicine, University Medical Center Hamburg-Eppendorf, Martinistr. 52, 20246 Hamburg, Germany; 2 Scanomed Nuclear Medicine Centers, Debrecen, Hungary

**Keywords:** Dopamine transporter, SPECT, Collimator, Pinhole, Between-rater agreement, Confidence

## Abstract

**Background:**

Multiple-pinhole (MPH) collimators improve the resolution–sensitivity trade-off compared to parallel-hole collimators. This study evaluated the impact of MPH collimators on intra- and between-rater agreement, and on the certainty of visual interpretation in dopamine transporter (DAT)-SPECT.

**Methods:**

The study included 71 patients (62.1 ± 12.7 y). Two SPECT acquisitions were performed in randomized order after a single injection of 182 ± 9 MBq ^123^I-FP-CIT, one with MPH and one with low-energy–high-resolution–high-sensitivity (LEHRHS) collimators. MPH projections were reconstructed with an iterative 3d Monte Carlo algorithm. LEHRHS projections were reconstructed with filtered backprojection (FBP) or with ordered-subsets expectation–maximization and resolution recovery (OSEM). Images were visually evaluated twice by three independent raters with respect to presence/absence of Parkinson-typical reduction of striatal ^123^I-FP-CIT uptake using a Likert 6-score (− 3 = clearly normal, …, 3 = clearly reduced). In case of intra-rater discrepancy, an intra-rater consensus was obtained. Intra- and between-rater agreement with respect to the Likert score (6-score and dichotomized score) was characterized by Cohen’s kappa.

**Results:**

Intra-rater kappa of visual scoring of MPH/LEHRHS-OSEM/LEHRHS-FBP images was 0.84 ± 0.12/0.73 ± 0.06/0.73 ± 0.08 (6-score, mean of three raters) and 1.00 ± 0.00/0.96 ± 0.04/0.97 ± 0.03 (dichotomized score). Between-rater kappa of visual scoring (intra-rater consensus) of MPH/LEHRHS-OSEM/LEHRHS-FBP images was 0.70 ± 0.06/0.63 ± 0.08/0.48 ± 0.05 (6-score, mean of three pairs of raters) and 1.00 ± 0.00/0.92 ± 0.04/0.90 ± 0.06 (dichotomized score). There was a decrease of (negative) Likert scores in normal DAT-SPECT by 0.87 ± 0.18 points from the LEHRHS-OSEM to the MPH setting. The (positive) Likert scores of reduced DAT-SPECT did not change on average.

**Conclusions:**

MPH collimators improve intra- and between-rater agreement as well as the certainty of the visual interpretation of DAT-SPECT.

**Supplementary Information:**

The online version contains supplementary material available at 10.1186/s13550-022-00923-w.

## Background

Single-photon emission computed tomography (SPECT) with the dopamine transporter (DAT) ligand *N*-ω-fluoropropyl-2β-carbomethoxy-3β-(4-I-123-iodophenyl)nortropane (^123^I-FP-CIT, trade name DaTSCAN, GE Healthcare) is widely used to support the etiological diagnosis of clinically uncertain Parkinsonian syndromes by providing evidence for (or against) loss of DAT in the striatum and its subregions [1–5].

The loss of striatal DAT in neurodegenerative Parkinsonian syndromes typically starts at the posterior putamen [6], a rather slim brain structure. As a consequence, the interpretation of DAT-SPECT can be affected by the limited spatial resolution of conventional SPECT imaging, particularly at early disease stages. Multiple-pinhole (MPH) collimator technology has the potential for concurrent improvement of both spatial resolution and count sensitivity compared to conventional imaging with parallel-hole and fan-beam collimators in clinical SPECT of small organs [7], including DAT-SPECT with ^123^I-FP-CIT [8–16].

The aim of this prospective study was the clinical evaluation of a novel general purpose brain imaging MPH collimator for its use in DAT-SPECT. SPECT with MPH collimators was compared with SPECT with low-energy–high-resolution–high-sensitivity (LEHRHS) collimators with respect to intra- and between-rater agreement and with respect to the certainty of the visual interpretation of DAT-SPECT.

## Materials and methods

### Patients

The prospective study included 71 patients (62.1 ± 12.7 years, range 34–85 years, 41% females) referred to DAT-SPECT in clinical routine to support the etiological diagnosis of a clinically uncertain Parkinsonian syndrome [1, 3, 17]. The study included only patients who were able to give informed consent and had sufficiently good health status so that a second SPECT acquisition immediately after the first was considered an acceptable burden to the patient. There were no further eligibility criteria in order to guarantee that the included patient sample was representative of clinical routine at our site.

There was only very limited clinical information available for the vast majority of the patients, because 56 out of 71 (79%) patients were referred to our department for DAT-SPECT by external neurologists. However, it can be assumed that the patient sample is representative of everyday clinical routine at the Department of Nuclear Medicine of the University Medical Center Hamburg-Eppendorf, since no specific eligibility criteria were imposed for this study. From previous studies in our department that included patients in whom clinical follow-up was available [18], it might be assumed that among the patients with reduced DAT-SPECT about 90% had a disease from the spectrum of Lewy body diseases (Parkinson’s disease without or with cognitive impairment, dementia with Lewy bodies), whereas the remaining 10% suffered from an atypical neurodegenerative Parkinsonian syndrome including multiple systems atrophy, progressive supranuclear palsy, and corticobasal degeneration. The diagnoses of the patients with normal DAT-SPECT most likely included essential tremor, drug-induced Parkinsonism, various types of dystonia, psychogenic Parkinsonism, and various other diagnoses not associated with nigrostriatal degeneration.

LEHRHS-SPECT and MPH-SPECT were performed in randomized order after a single injection of 182 ± 9 MBq ^123^I-FP-CIT (range 163–200 MBq). Both SPECT acquisitions were performed with the same general purpose triple-head SPECT camera (AnyScan Trio SC, Mediso Medical Imaging Systems, Budapest, Hungary) in order to avoid ‘contamination’ of collimator effects by camera effects.

### MPH-SPECT

The general purpose brain MPH collimator tested in this study was designed for high count sensitivity at the center of the field of view with a rather broad peak of the sensitivity profile for improved stability with respect to off-center positioning (e.g., of the striatum). The collimator features a solid tungsten aperture of 18 mm thickness with 30 pinholes arranged in 5 axially oriented columns and 6 transaxially oriented rows. The MPH aperture is mounted on a lead blind that defines the orthogonal distance between the pinhole focal plane and the detector surface to 145 mm.

A total of 90 projection views (30 per head, 120° scan arc) at angular steps of 4° were acquired in a 256 × 256 matrix with 2.13 mm × 2.13 mm pixel size. The energy window was set to 143–175 keV. The distance between the center-of-rotation axis and the pinhole focal plane was fixed to 140 mm. Helical acquisition mode was used to avoid axial undersampling [19, 20]. Helical acquisition was achieved by moving the patient table at each angular gantry step out of the gantry. The total table displacement during the SPECT acquisition was 40 mm. The total (net) duration of the MPH acquisition was 30 min.

MPH projection data were reconstructed to transaxial SPECT images with the Monte Carlo photon simulation engine and iterative one-step-late maximum-a-posteriori expectation–maximization implemented in the camera software (30 iterations, 3 subsets). A more detailed description of the reconstruction method has been given previously [16, 21]. Chang’s order zero method with linear broad-beam attenuation coefficient *μ* = 0.12/cm was used for post-reconstruction attenuation correction [22]. Scatter correction was not performed.

### LEHRHS-SPECT

LEHRHS-SPECT was performed in double-head mode, that is, using only two of the three detector heads. The third head was switched off and moved away from the center of rotation in order to allow about 140 mm radius of rotation with the two remaining detectors. Interlaced triple-head mode [23] was not available for the SPECT camera used in this study. A total of 120 projection views (60 per head, 180° scan arc) at angular steps of 3° were acquired in a 128 × 128 matrix with 2.43 mm × 2.43 mm pixel size. The energy window was set to 143–175 keV. The radius of rotation was 146 ± 7 mm. The total (net) duration of the LEHRHS acquisition was 40 min.

Two different algorithms were used for the reconstruction of the LEHRHS projection data. First, transaxial SPECT images were obtained by filtered backprojection (FBP) implemented in the SPECT camera software (Butterworth window of 6th order and 2.3 cycles/cm cutoff). Uniform post-reconstruction attenuation correction was performed according to order zero Chang (*µ* = 0.12/cm), no scatter correction. Second, SPECT images were reconstructed using the iterative ordered-subsets expectation–maximization algorithm with resolution recovery implemented in the HybridRecon-Neurology tool of the Hermes SMART workstation v1.6 with parameter settings recommended for FP-CIT SPECT by Hermes (5 iterations, 15 subsets, post-filtering with 3-dimensional Gaussian kernel of 7 mm full width at half maximum, uniform attenuation correction with narrow-beam attenuation coefficient 0.146/cm, simulation-based scatter correction, resolution recovery with a Gaussian model).

**Fig. 1:**
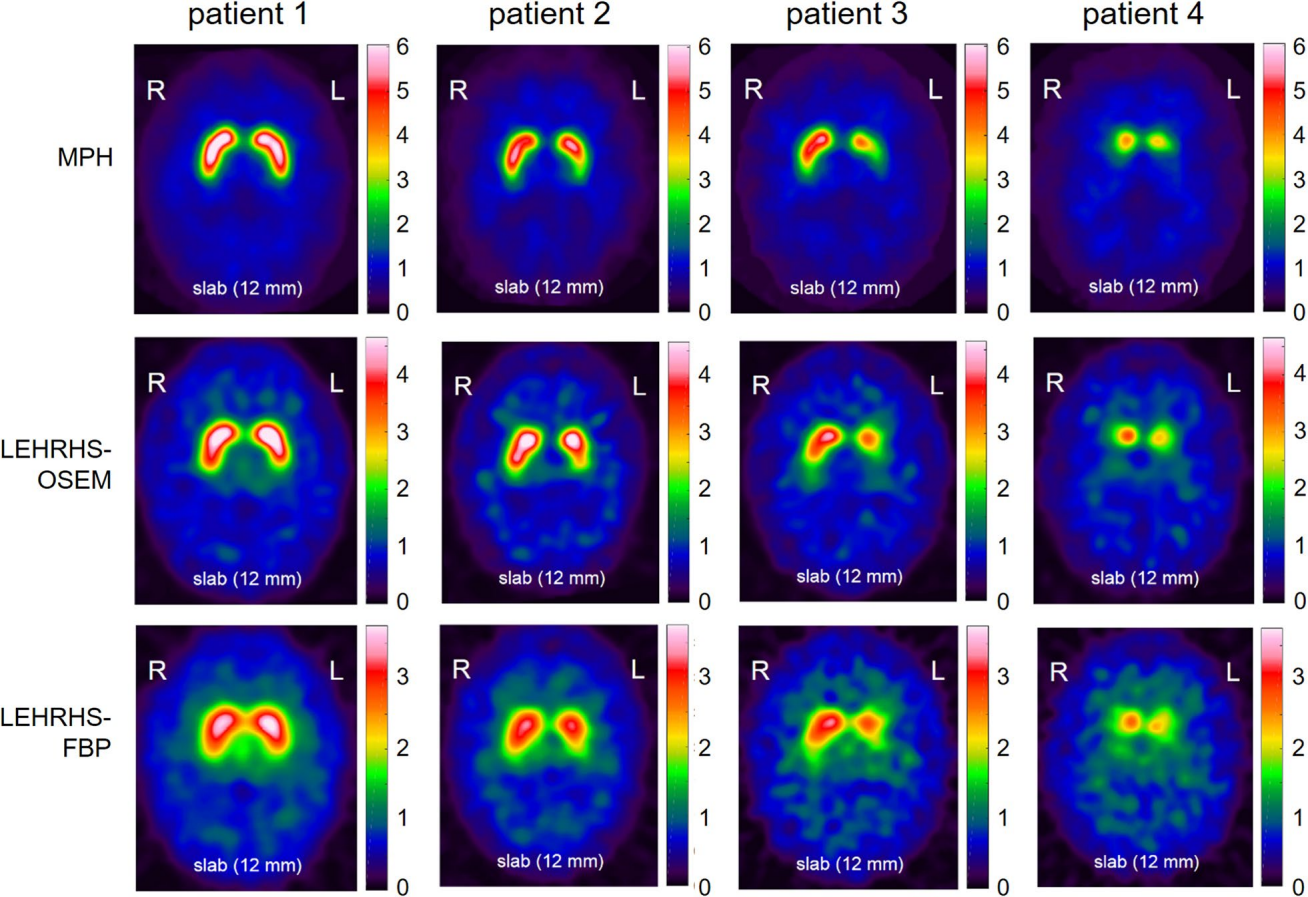
Representative DAT-SPECT images. Representative DAT-SPECT images from four different patients (columns) in the three different settings (rows)

Representative SPECT images are shown in Fig. 1.

The SPECT acquisition was performed first with the MPH collimators in 46 of the 71 patients (65%). The mean delay of the start of the subsequent acquisition with LEHRHS collimators relative to the start of the MPH acquisition was 53 ± 8 min. The SPECT acquisition was performed first with the LEHRHS collimators in the remaining 25 patients (35%). The mean delay of the start of the subsequent acquisition with MPH collimators relative to the start of the LEHRHS acquisition was 63 ± 7 min. (Note that the net duration of the acquisition was 10 min longer with the LEHRHS collimators, 40 min versus 30 min.) The delay between i.v. administration of ^123^I-FP-CIT and start of the acquisition was 212 ± 39 min for the MPH acquisitions and 224 ± 37 min for the LEHRHS acquisitions (paired *t* test *p* = 0.067).

### Visual interpretation

**Fig. 2:**
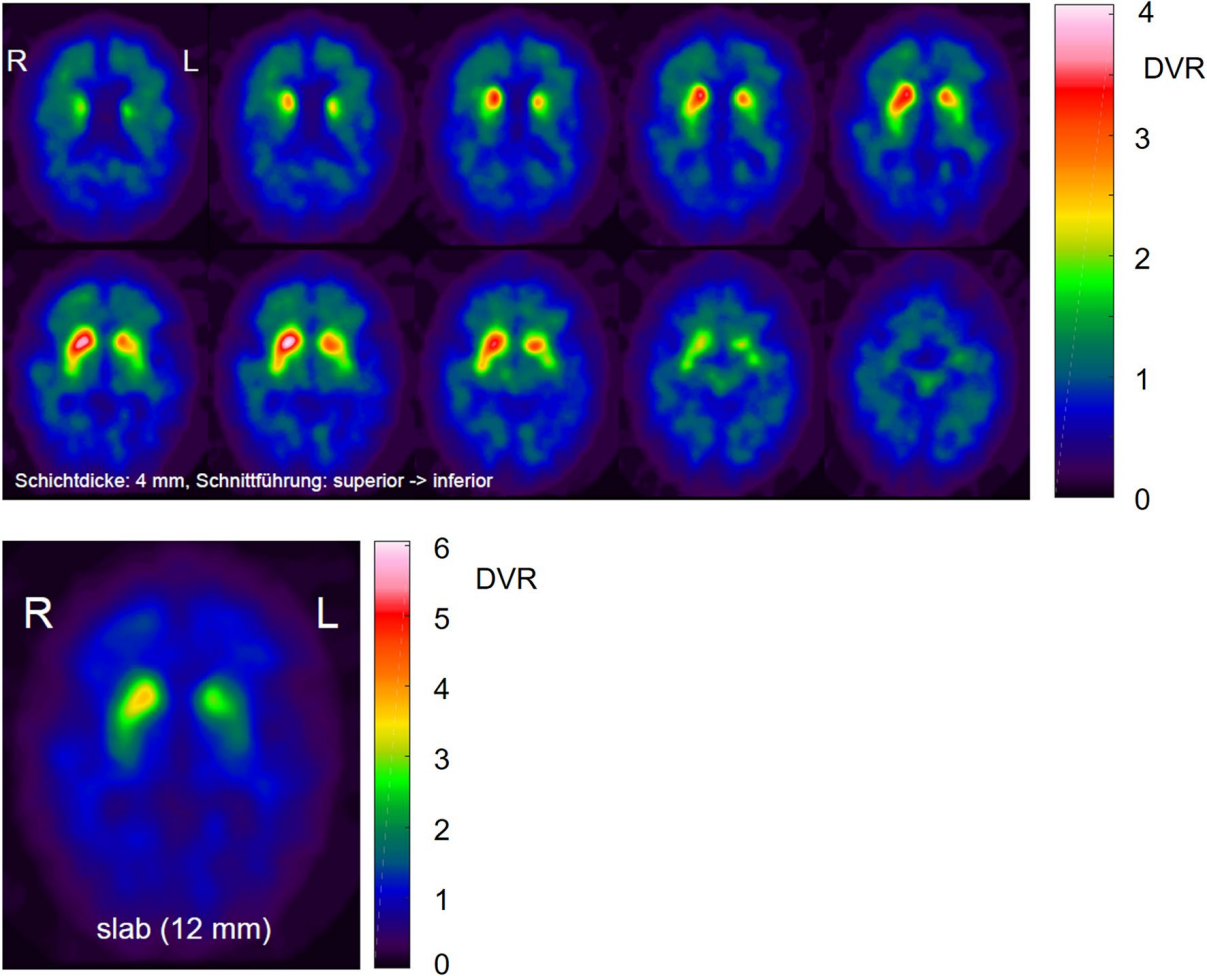
Standard display for visual interpretation of the DAT-SPECT images. Standard display for visual interpretation of the DAT-SPECT images presenting ten transversal distribution volume ratio (DVR) image slices of 4 mm thickness from the superior to the inferior edge of the striatum with the maximum of the colortable individually scaled to the maximum intensity in the ten images. In addition, the display presents a transversal DVR image slab of 12 mm thickness through the center of the striatum with the maximum of the colortable scaled to a fixed DVR threshold [25]. The DVR threshold had been optimized previously, separately for each of the three settings (MPH, LEHRH-FBP, LEHRHS-OSEM)

Visual interpretation of the DAT-SPECT images was based on a standardized display (Fig. 2), similar to the display used in everyday clinical routine in our department. For generation of the display, each individual DAT-SPECT image was stereotactically normalized (affine transformation) to the anatomical standard space of the Montreal Neurological Institute (MNI) using the Normalization tool of the Statistical Parametric Mapping software package (version SPM12) and a custom FP-CIT template. The stereotactically normalized DAT-SPECT image was smoothed by convolution with an isotropic Gaussian kernel with 4 mm full width at half maximum, independent of the collimator used for the SPECT acquisition. Distribution volume ratio (DVR) images were obtained by voxelwise scaling of the smoothed image to the 75th percentile of the intensity values in a reference region comprising the whole brain parenchyma without striata, thalamus and brainstem [24]. The display comprised ten transversal DVR image slices of 4 mm thickness and one transversal DVR image slab of 12 mm thickness (Fig. 2) [25].

Visual interpretation of the SPECT images was performed independently by three raters with different experience in clinical reading of DAT-SPECT (about 200, about 2000, and about 5000 cases). The raters were blinded for all clinical data. The raters were asked to score each SPECT image with respect to Parkinson-typical reduction of striatal ^123^I-FP-CIT uptake using the following Likert 6-score: − 3 = clearly normal, − 2 = probably normal, − 1 = more likely normal than reduced, 1 = more likely reduced than normal, 2 = probably reduced, 3 = clearly reduced. This was performed twice for each of the three settings (MPH, LEHRHS-FBP, LEHRHS-OSEM); that is, each rater performed six reading sessions. A different randomization of the patients was used in each session. Images with a discrepant Likert score between the two reading sessions of a given setting by a given rater were rated a third time by the same rater to obtain an intra-rater consensus.

### Semiquantitative analysis

The specific binding ratio (SBR) of ^123^I-FP-CIT in left and right putamen was obtained by hottest voxels analysis of the stereotactically normalized DVR image using large unilateral putamen masks predefined in MNI space [18]. The putamen masks were much bigger than the actual putamen volume to guarantee that all putaminal counts were included. The number of hottest voxels within a unilateral putamen mask to be averaged was fixed to a total volume 10 ml. The putamen SBR was computed as mean DVR in the hottest voxels minus 1. The minimum putamen SBR of both hemispheres was used for the analysis.

### Statistical analysis

Continuous variables are reported as mean ± standard deviation of the sample. Intra- and between-rater agreement with respect to the Likert score and with respect to the dichotomized Likert score (< 0: normal, > 0: reduced) was characterized by Cohen’s kappa. Cohen’s effect size d was used to characterize the difference of the putamen SBR between visually normal and visually reduced DAT-SPECT.

Statistical analyses were conducted using SPSS version 27 (SPSS Inc., Chicago, Illinois). All p-values are given two-sided. Statistical significance was defined as *p* < 0.05.

### Technical performance characteristics of MPH-SPECT

The system count sensitivity profile of the triple-head SPECT system equipped with the novel general purpose brain imaging MPH collimator was measured as described previously by our group for a DAT-SPECT-specific MPH collimator [16]. In brief, a point source (5–10 MBq ^99m^Tc in about 5 µl in an Eppendorf tube) was placed on the lattice points of a 1 cm grid covering the whole field of view. A full SPECT acquisition was performed for each localization of the point source using the same acquisition parameters as for clinical DAT-SPECT described in ‘MPH-SPECT’ section. The total number of counts acquired during the SPECT acquisition was obtained by summing the counts over all views. Uniformity correction was turned on. Dead time correction was negligible. The system count sensitivity at the localization of the point source was obtained by the following formula: sensitivity = total number of counts/total net scan duration/activity of the point source decay-corrected to the start time of the SPECT acquisition.

Spatial resolution of MPH-SPECT was assessed with a Derenzo-type hot rod phantom with rod diameter of 2 to 7 mm filled with ^123^I solution. Acquisition and image reconstruction were performed using exactly the same protocol and parameter settings as for clinical MPH-SPECT except that no attenuation correction was performed.

## Results

### Intra- and between-rater variability of visual interpretation

The cross-tables with respect to intra- and between-rater agreement are given in the additional material (Additional file 1: Tables S1–S6).

**Fig. 3:**
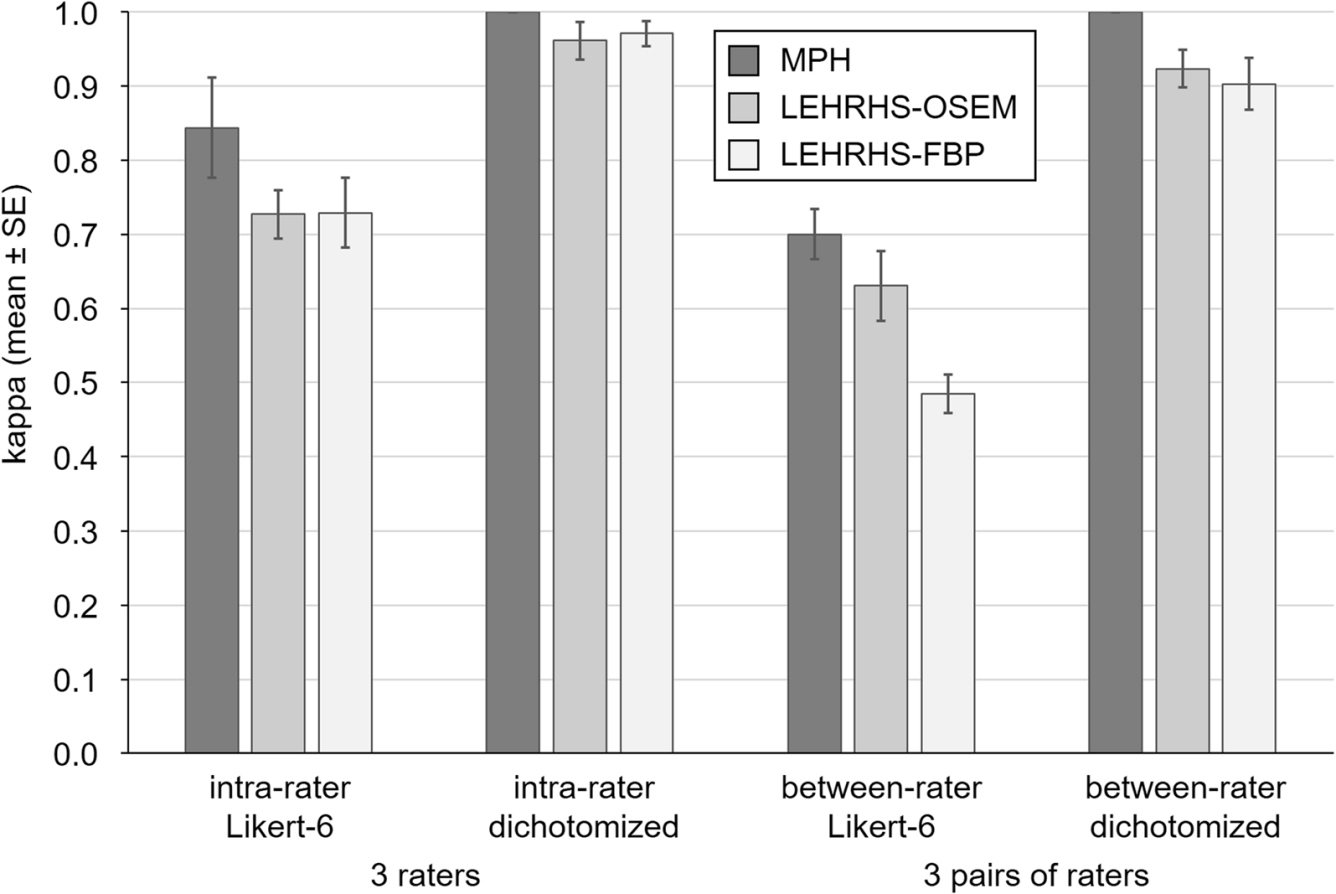
Intra- and between-rater variability. Intra- and between-rater variability of the visual interpretation of the DAT-SPECT images according to the Likert 6-score and according to the dichotomized Likert 6-score averaged over the three individual raters, respectively, the 3 pairs of raters. (SE = standard error of the mean)

Intra-rater kappa of visual scoring of MPH/LEHRHS-OSEM/LEHRHS-FBP images was 0.84 ± 0.12/0.73 ± 0.06/0.73 ± 0.08 (mean ± standard deviation of the three raters) with respect to the full Likert 6-score and 1.00 ± 0.00/0.96 ± 0.04/0.97 ± 0.03 with respect to the dichotomized score (Fig. 3; intra-rater kappa values for each individual rater are given in Additional file 1: Fig. S1). Between-rater kappa of visual scoring (intra-rater consensus) of MPH/LEHRHS-OSEM/LEHRHS-FBP images was 0.70 ± 0.06/0.63 ± 0.08/0.48 ± 0.05 (mean ± standard deviation of the three pairs of raters) with respect to the full Likert 6-score and 1.00 ± 0.00/0.92 ± 0.04/0.90 ± 0.06 with respect to the dichotomized score (Fig. 3; between-rater kappa values for each pair of raters are given in Additional file 1: Fig. S1).

Changes of the intra-rater consensus Likert 6-score between LEHRHS-FBP and LEHRHS-OSEM and between LEHRHS-OSEM and MPH, separately for each individual rater, are shown in Additional file 1: Fig. S2.

The dichotomized Likert score in the MPH setting showed perfect intra-rater and between-rater agreement (all kappa = 1; Fig. 3, Additional file 1: Fig. S1). Thus, the dichotomized Likert score in the MPH setting was used as standard of truth for ‘normal’ (*n* = 31) or ‘reduced’ (*n* = 40) DAT-SPECT in the following analyses.

**Fig. 4:**
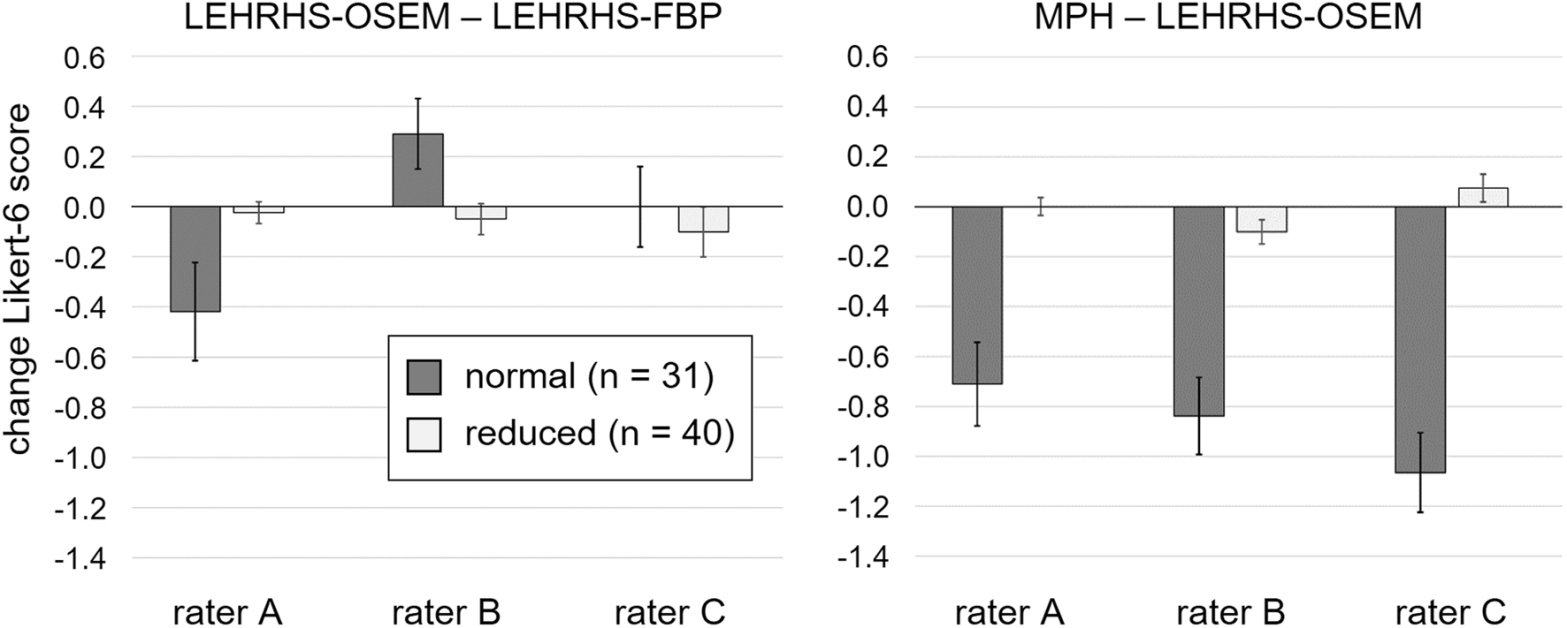
Changes of the intra-rater consensus Likert 6-score. Changes of the intra-rater consensus Likert 6-score between LEHRHS-FBP and LEHRHS-OSEM (left) and between LEHRHS-OSEM and MPH (right), separately for normal and reduced DAT-SPECT

Changes of the intra-rater consensus Likert 6-score between LEHRHS-FBP and LEHRHS-OSEM and between LEHRHS-OSEM and MPH are shown in Fig. 4, separately for normal and reduced DAT-SPECT.

### Semiquantitative analysis

**Fig. 5:**
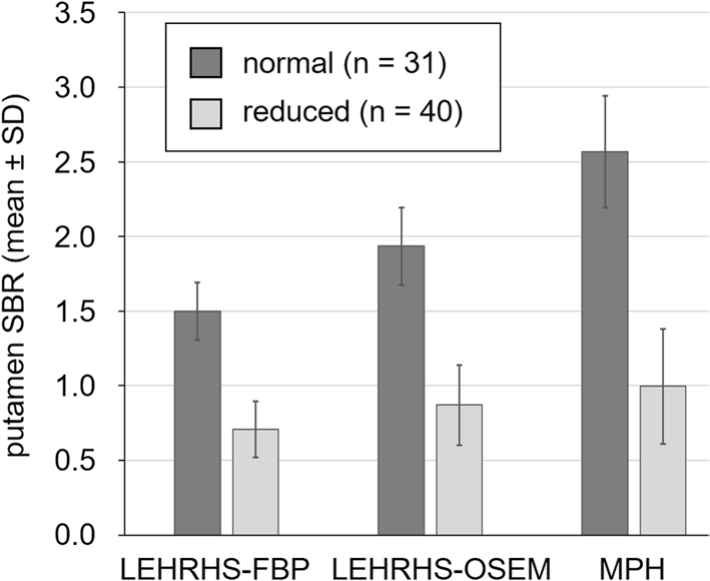
SBR of ^123^I-FP-CIT in the putamen. SBR of ^123^I-FP-CIT in the putamen (minimum of both hemispheres) in normal versus reduced DAT-SPECT, separately for the 3 settings. (SD = standard deviation)

The putamen SBR of ^123^I-FP-CIT in normal DAT-SPECT compared to reduced DAT-SPECT is shown in Fig. 5, separately for the three settings. Cohen’s effect size of the difference between normal and reduced DAT-SPECT was 4.2, 4.1, and 4.2 in the LEHRHS-FBP, the LEHRHS-OSEM, and in the MPH setting, respectively.

### Technical performance characteristics of MPH-SPECT

**Fig. 6:**
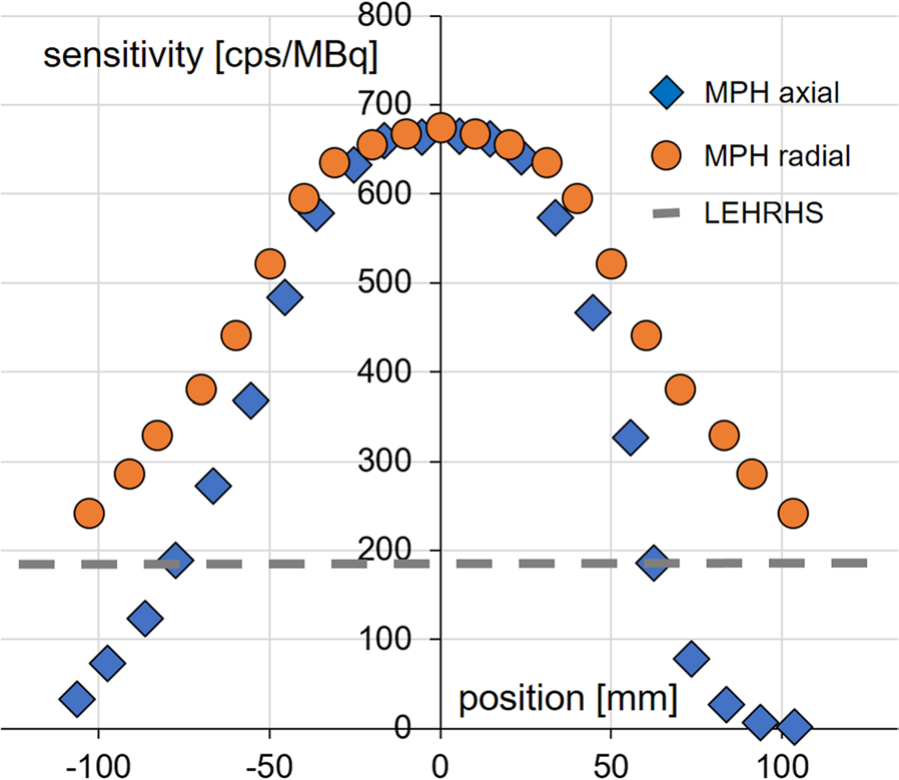
Count sensitivity profiles. System count sensitivity profile of the triple-head SPECT system with the MPH collimators in axial direction (*z*-direction) and in radial (transaxial) direction compared to the uniform system sensitivity of the SPECT system in double-head mode with LEHRHS collimators. The radial sensitivity profile was measured at the maximum of the axial profile

System count sensitivity profiles of the triple-head SPECT system with MPH collimators are shown in Fig. 6. Peak system sensitivity was about 675 cps/MBq compared to about 190 cps/MBq for the SPECT system in double-head mode with LEHRHS collimators. System sensitivity with the MPH collimators decreased toward the edges of the field of view. The full width of the axial sensitivity profile with the MPH collimators at 190 cps/MBq (= sensitivity in double-head mode with the LEHRHS collimator) was about 140 mm. This is sufficient to fully cover the whole brain (including the cerebellum) in inferior–superior direction in the vast majority of patients [26]. The radial system sensitivity profile was considerably broader than the axial profile (Fig. 6) and, therefore, easily covers the full brain in left–right direction and in anterior–posterior direction in all patients [26].

**Fig. 7:**
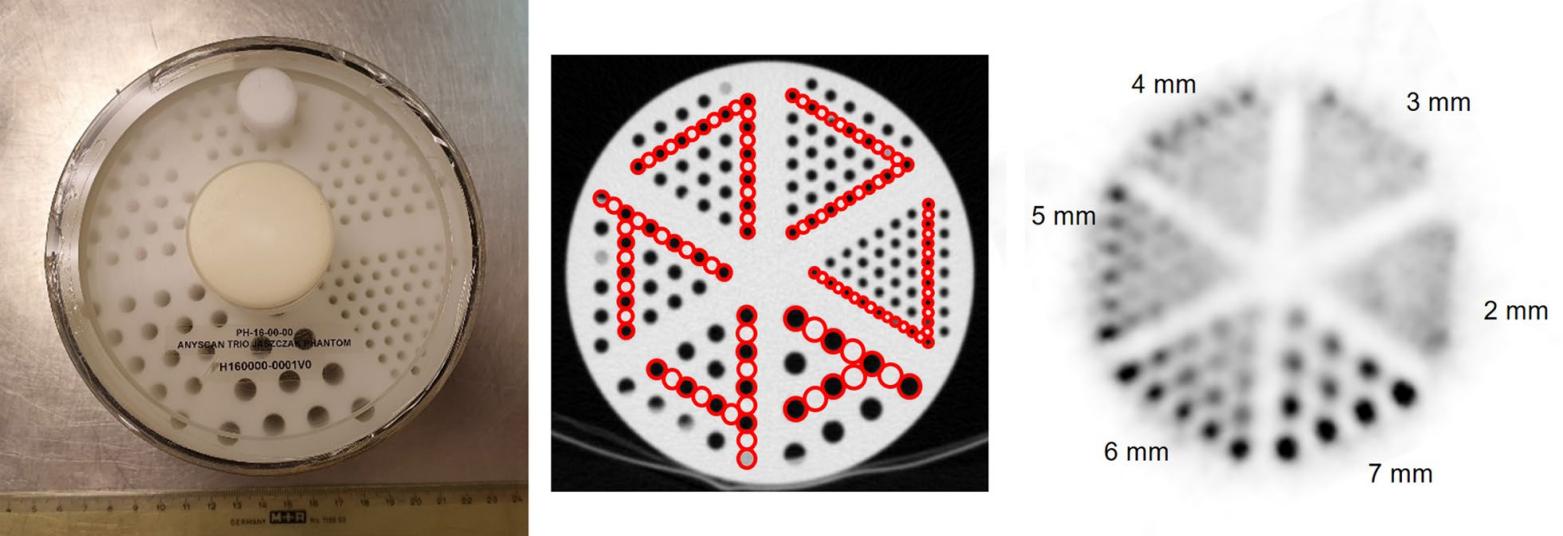
SPECT with MPH collimators of a Derenzo-type hot rod phantom. Transaxial SPECT image (right) of a Derenzo-type hot rod phantom (left) filled with ^123^I solution that was acquired with the MPH collimators using the same acquisition protocol and the same reconstruction parameter settings as for clinical DAT-SPECT except that attenuation correction was not performed. The red circles overlaid to a CT image of the phantom (middle) illustrate that the distance between neighboring rods equals their diameter

A transversal slice of the Derenzo-type hot rod phantom acquired with MPH-SPECT is shown in Fig. 7. The rods with ≥ 6 mm diameter were clearly visible, and the rods with ≤ 4 mm diameter could not be detected reliably. The rods with 5 mm diameter were visible, but with low contrast.

## Discussion

The aim of this prospective study was the evaluation of a novel general purpose brain imaging MPH collimator with respect to intra- and between-rater agreement and with respect to the certainty of the visual interpretation of DAT-SPECT. The MPH collimator had not been specifically designed for DAT-SPECT but for all brain SPECT applications including brain perfusion SPECT.

The primary finding of this study is that DAT-SPECT with the brain MPH collimator improves intra- and between-rater agreement, and the certainty of the visual interpretation of the SPECT images compared to DAT-SPECT with a conventional LEHRHS collimator (Fig. 3). The improvement of intra-rater agreement was observed for each individual rater (Additional file 1: Fig. S1), suggesting that it is not restricted to less experienced raters (after clinical reading of about 200 DAT-SPECT), but also experienced (about 2000 DAT-SPECT) and very experienced (about 5000 DAT-SPECT) raters benefit from the improved image quality.

In clinical routine, interpretation of DAT-SPECT is restricted to the binary decision as ‘reduced’ (indicating nigrostriatal degeneration) or ‘normal’ (indicating a secondary Parkinsonian syndrome without nigrostriatal degeneration). It is particularly relevant, therefore, that the dichotomized Likert 6-score showed perfect intra- and between-rater agreement for each individual rater and for each pair of raters with the MPH collimators only (Additional file 1: Fig. S1). With the LEHRHS-SPECT images, the raters still achieved very good intra-rater and between-rater agreement (kappa about 0.95 and about 0.90, respectively), but not perfect. There was no clear difference between LEHRHS-OSEM and LEHRHS-FBP to favor one over the other with respect to intra- and between-rater agreement of the visual interpretation, although iterative reconstruction with attenuation and scatter correction and resolution recovery provided better image quality (Fig. 1), in line with previous reports [27].

**Fig. 8:**
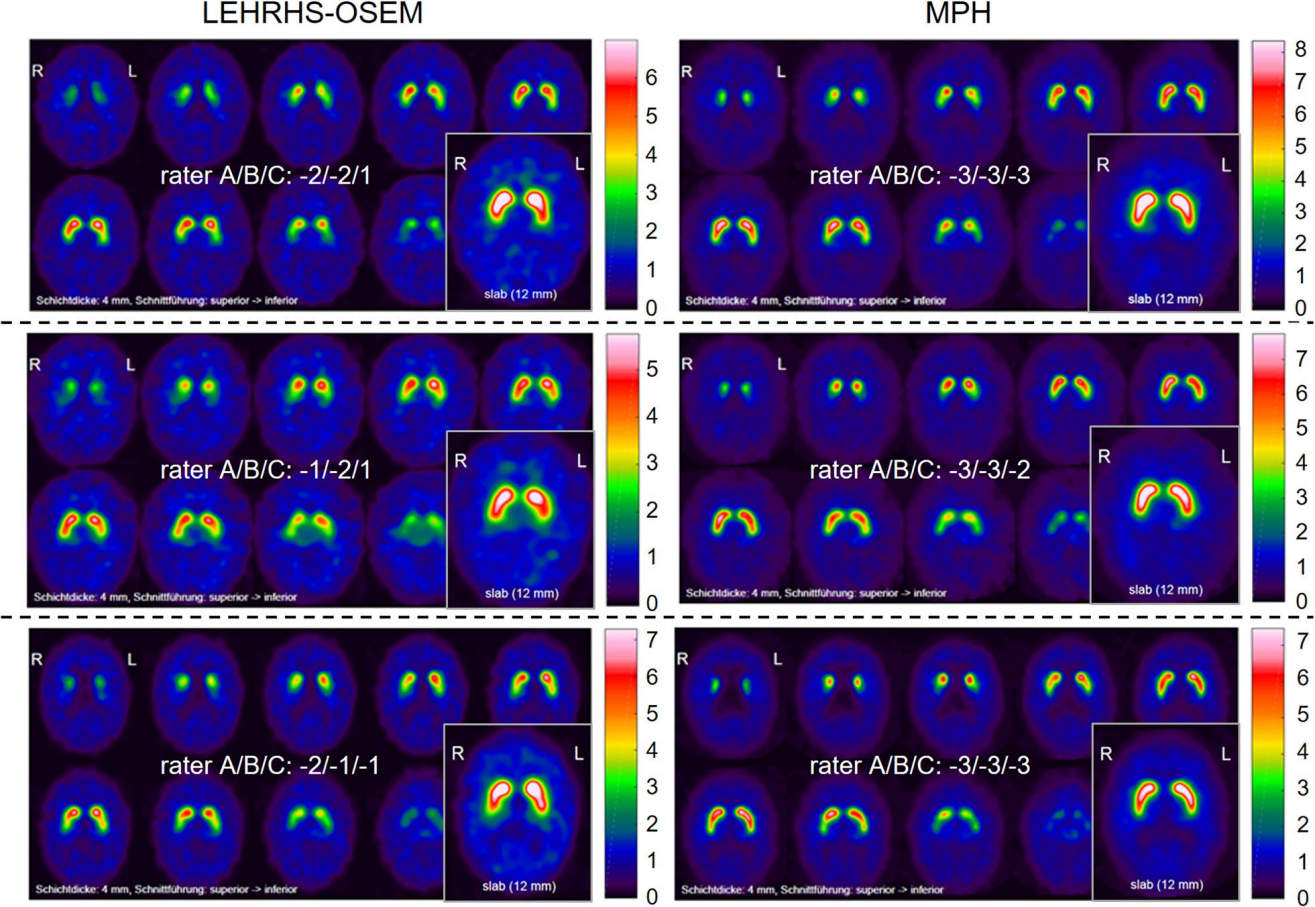
Examples of improved certainty. Three different patients (one patient per row) with improved certainty of the visual interpretation with MPH-SPECT compared to LEHRHS-SPECT (iterative reconstruction)

The improvement of rater confidence in the visual interpretation of DAT-SPECT by MPH collimators was mainly driven by improved confidence in excluding nigrostriatal degeneration (Fig. 4, Additional file 1: Fig. S2). This might be explained by the fact that DAT loss associated with nigrostriatal degeneration in neurodegenerative Parkinsonian syndromes typically starts in the posterior part of the putamen, which is thinner than its anterior part. Thus, the posterior part of the putamen is particularly prone to partial volume effects caused by limited spatial resolution in the SPECT images. Improved spatial resolution of MPH-SPECT results in less pronounced partial volume effects and, consequently, more reliable depiction of the posterior putamen (Fig. 8). The impact of MPH-SPECT on the confidence of the detection of nigrostriatal degeneration was small (Fig. 4, Additional file 1: Fig. S2).

We hypothesize that the improvement of image quality with respect to both spatial resolution and statistical noise by MPH-SPECT also improves the confidence of the referring neurologist in DAT-SPECT findings, because the improved image quality with better anatomical delineation of the striatum and its substructures (i) makes the SPECT images more appealing for readers more familiar with structural MRI and (ii) makes it easier for non-experts to comprehend their interpretation by the nuclear medicine physician.

Improved spatial resolution of MPH-SPECT resulted in considerably higher estimates of the putamen SBR compared to LEHRHS-SPECT (Fig. 5). However, the effect was similar in normal and reduced DAT-SPECT so that the effect size of the difference between normal and reduced DAT-SPECT was practically the same in all settings. Thus, the impact of MPH collimators on semiquantitative analysis in DAT-SPECT most likely is small. This might be explained by the fact that averaging intensities of a rather large number of voxels in the striatal region of interest (or in the reference region) levels out differences with respect to spatial resolution and statistical noise to large extent.

However, there are early signs of Parkinson’s disease such as smell loss and rapid eye movement sleep and behavior disorder that precede motor symptoms. As soon as disease-modifying drugs for treatment of Parkinson’s disease will be available, it will be important to detect Parkinson’s disease also in these early stages, when the loss of (unilateral) putaminal DAT is considerably below the 50% threshold for the occurrence of motor symptoms [28–30]. MPH collimators might be particularly useful to improve sensitivity and specificity for the detection of mild nigrostriatal degeneration at early (pre-motor) stages of the disease.

The following limitations of the study should be noted. First, potential impact of MPH-SPECT on the diagnostic accuracy could not be assessed, because there was no independent gold standard diagnosis available. All included patients were referred to DAT-SPECT to support the etiological diagnosis of a clinically uncertain Parkinsonian syndrome. Thus, a sufficiently reliable clinical diagnosis was not available. However, among the 67 of the 71 patients in which the three raters agreed with respect to the binary classification of the iteratively reconstructed LEHRHS-SPECT images there was no one with different binary classification from the MPH-SPECT images. This suggests that the impact of the brain MPH collimators on the binary classification of DAT-SPECT is small. Second, the patient sample was slightly unbalanced with respect to the ordering of the SPECT acquisition. MPH-SPECT was performed first in about two-thirds of the cases. As a consequence, there might have been a bias in favor of MPH-SPECT, as head motion might have been more frequent during the second acquisition due to possible exhaustion of patients. The impact of the difference in the delay between injection of ^123^I-FP-CIT and the start of the acquisition between MPH-SPECT and LEHRHS-SPECT most likely was small, since all acquisitions were performed within the recommended time window 3–6 h after injection [4, 31]. Third, MPH-SPECT images were reconstructed without scatter correction despite the fact that about 2.5% high-energy (≥ 440 keV) photons emitted by ^123^I cause scatter counts in the standard energy window centered at 159 keV, strongly depending on the collimator [32]. The potential impact of scatter correction on the performance of DAT-SPECT with MPH collimators should be evaluated in further studies. Finally, practice guidelines on DAT-SPECT recommend the use of fan-beam collimators [4], which provide a 20–40% gain in count sensitivity at similar spatial resolution compared to LEHRHS collimators [7, 33]. There are no fan-beam collimators available for the triple-head SPECT system used in this study. We decided not to use a different SPECT camera equipped with fan-beam collimators in order to avoid ‘contamination’ of collimator effects by camera effects.

In conclusion, MPH collimators improve intra- and between-rater agreement as well as the certainty of the visual interpretation in DAT-SPECT, particularly for the exclusion of nigrostriatal degeneration, and therefore can be recommended for routine clinical use.

## Supplementary Information

**Additional file 1.** Additional Tables and Figures. Additional Tables S1–S6 and Additional Figures S1, S2.

## Data Availability

All data used in this study can be made available in anonymized form on request.

## Abbreviations

^123^I-FP-CIT: *N*-ω-Fluoropropyl-2β-carbomethoxy-3β-(4-I-123-iodophenyl)nortropane
*d*: Cohen’s effect size
DAT: Dopamine transporter
DVR: Distribution volume ratio
FBP: Filtered backprojection
LEHRHS: Low-energy–high-resolution–high-sensitivity
MNI: Montreal Neurological Institute
MPH: Multiple-pinhole
OSEM: Ordered-subsets-expectation–maximization
SBR: Specific binding ratio
SPECT: Single-photon emission computed tomography
SPM: Statistical parametric mapping
