# Supplementary material for: Multiple-pinhole collimators improve intra- and between-rater agreement and the certainty of the visual interpretation in dopamine transporter SPECT
Source: EJNMMI Res. 2022 Aug 17;12:51. doi: 10.1186/s13550-022-00923-w (PMC9385910; doi:10.1186/s13550-022-00923-w)
Supplement: Supplementary file 1 — Additional file 1. Additional Tables and Figures. Additional Tables S1–S6 and Additional Figures S1, S2. [file 13550_2022_923_MOESM1_ESM.docx]

**Online supplementary**

**Multiple-pinhole collimators improve intra- and between-rater agreement and the
certainty of the visual interpretation in dopamine transporter SPECT**

Franziska Mathies^1^, Ivayla Apostolova^1^, Lena Dierck^1^, Janin Jacobi^1^, Katja Kuen^1^, Markus Sauer^1^, Michael Schenk^1^, Susanne Klutmann^1^, Attila Forgács^2^, Ralph Buchert^1^

^1^Department of Diagnostic and Interventional Radiology and Nuclear Medicine, University Medical Center Hamburg-Eppendorf, Hamburg, Germany

^2^Scanomed Nuclear Medicine Centers, Debrecen, Hungary

**Corresponding author:** Ralph Buchert, Department of Diagnostic and Interventional Radiology and Nuclear Medicine, University Medical Center Hamburg-Eppendorf, Martinistr. 52, 20246 Hamburg, Germany, Email: [r.buchert@uke.de](mailto:r.buchert@uke.de), Phone: +49 (0)40 7410-54347, Fax: +49 (0)40 7410-40265, ORCID ID 0000-0002-0945-0724

**Supplementary Tables**

Supplementary tables S1-S3 provide cross tables with respect to intra-rater agreement, supplementary tables S4-S6 with respect to between-rater agreement of the visual interpretation of the 71 DAT-SPECT images according to the Likert 6-score (-3=clearly normal, …, 3=clearly reduced) for the 3 different settings (MPH, LEHRHS-OSEM, LEHRHS-FBP). (blue = consistent scores, orange = discrepant scores with respect to the Likert 6-score but consistent with respect to the binary categorization as normal or reduced, red = discrepant scores with respect to the binary categorization as normal or reduced)

**Supplementary Table S1** Intra-rater agreement of rater A

| **Likert**  **6-score** | | **second reading session** | | | | | | | | | | | | | | | | | |
| --- | --- | --- | --- | --- | --- | --- | --- | --- | --- | --- | --- | --- | --- | --- | --- | --- | --- | --- | --- |
|  |  | **MPH** | | | | | | **LEHRHS-OSEM** | | | | | | **LEHRHS-FBP** | | | | | |
|  |  | **-3** | **-2** | **-1** | **1** | **2** | **3** | **-3** | **-2** | **-1** | **1** | **2** | **3** | **-3** | **-2** | **-1** | **1** | **2** | **3** |
| **first reading session** | **-3** | **25** | **1** |  |  |  |  | **8** | **2** |  |  |  |  | **4** | **1** |  |  |  |  |
|  | **-2** |  | **5** |  |  |  |  | **4** | **11** | **1** |  |  |  |  | **16** | **1** |  |  |  |
|  | **-1** |  |  |  |  |  |  |  | **2** | **2** |  |  |  |  | **3** | **2** |  |  |  |
|  | **1** |  |  |  |  |  | **1** |  |  |  | **2** | **1** |  |  |  |  | **2** | **1** |  |
|  | **2** |  |  |  |  | **2** |  |  |  |  |  |  | **1** |  |  |  | **1** | **1** |  |
|  | **3** |  |  |  |  |  | **37** |  |  |  |  |  | **37** |  |  |  |  | **1** | **38** |

**Supplementary Table S2** Intra-rater agreement of rater B

| **Likert**  **6-score** | | **second reading session** | | | | | | | | | | | | | | | | | |
| --- | --- | --- | --- | --- | --- | --- | --- | --- | --- | --- | --- | --- | --- | --- | --- | --- | --- | --- | --- |
|  |  | **MPH** | | | | | | **LEHRHS-OSEM** | | | | | | **LEHRHS-FBP** | | | | | |
|  |  | **-3** | **-2** | **-1** | **1** | **2** | **3** | **-3** | **-2** | **-1** | **1** | **2** | **3** | **-3** | **-2** | **-1** | **1** | **2** | **3** |
| **first reading session** | **-3** | **19** | **3** |  |  |  |  | **7** | **6** |  |  |  |  | **11** | **4** |  |  |  |  |
|  | **-2** | **4** | **2** | **2** |  |  |  | **2** | **4** |  |  |  |  | **2** | **7** |  |  |  |  |
|  | **-1** | **1** |  |  |  |  |  |  | **2** | **7** |  |  |  |  | **3** | **1** |  |  |  |
|  | **1** |  |  |  | **1** |  | **1** |  |  | **3** |  | **2** |  |  |  | **2** | **1** | **1** |  |
|  | **2** |  |  |  | **1** | **3** |  |  |  |  |  |  |  |  |  |  |  | **1** | **3** |
|  | **3** |  |  |  |  | **1** | **33** |  |  |  |  | **1** | **37** |  |  |  |  | **1** | **34** |

**Supplementary Table S3** Intra-rater agreement of rater C

| **Likert**  **6-score** | | **second reading session** | | | | | | | | | | | | | | | | | |
| --- | --- | --- | --- | --- | --- | --- | --- | --- | --- | --- | --- | --- | --- | --- | --- | --- | --- | --- | --- |
|  |  | **MPH** | | | | | | **LEHRHS-OSEM** | | | | | | **LEHRHS-FBP** | | | | | |
|  |  | **-3** | **-2** | **-1** | **1** | **2** | **3** | **-3** | **-2** | **-1** | **1** | **2** | **3** | **-3** | **-2** | **-1** | **1** | **2** | **3** |
| **first reading session** | **-3** | **21** | **2** |  |  |  |  | **2** | **2** |  |  |  |  | **1** | **1** |  |  |  |  |
|  | **-2** | **3** | **3** |  |  |  |  |  | **14** | **1** |  |  |  | **1** | **10** | **3** |  |  |  |
|  | **-1** |  |  | **2** |  |  |  |  | **5** | **5** | **1** |  |  |  | **7** | **8** | **1** |  |  |
|  | **1** |  |  |  | **1** |  |  |  |  |  | **2** | **1** |  |  |  |  |  |  |  |
|  | **2** |  |  |  |  | **1** | **1** |  |  |  |  |  | **1** |  |  |  |  |  |  |
|  | **3** |  |  |  |  |  | **37** |  |  |  |  |  | **37** |  |  |  |  |  | **39** |

**Supplementary Table S4** Between-rater agreement of rater A and rater B

| **Likert**  **6-score** | | **rater B** | | | | | | | | | | | | | | | | | |
| --- | --- | --- | --- | --- | --- | --- | --- | --- | --- | --- | --- | --- | --- | --- | --- | --- | --- | --- | --- |
|  |  | **MPH** | | | | | | **LEHRHS-OSEM** | | | | | | **LEHRHS-FBP** | | | | | |
|  |  | **-3** | **-2** | **-1** | **1** | **2** | **3** | **-3** | **-2** | **-1** | **1** | **2** | **3** | **-3** | **-2** | **-1** | **1** | **2** | **3** |
| **rater A** | **-3** | **23** | **2** |  |  |  |  | **9** |  | **1** |  |  |  | **3** | **2** |  |  |  |  |
|  | **-2** | **2** | **4** |  |  |  |  | **1** | **8** | **7** |  |  |  | **11** | **6** | **1** | **1** |  |  |
|  | **-1** |  |  |  |  |  |  |  | **2** | **2** |  |  |  | **1** | **2** |  |  |  |  |
|  | **1** |  |  |  |  |  |  |  |  | **1** | **1** |  |  |  | **1** | **3** |  |  |  |
|  | **2** |  |  |  | **2** |  | **1** |  |  |  | **1** |  |  |  |  |  |  | **1** | **1** |
|  | **3** |  |  |  |  | **4** | **33** |  |  |  |  |  | **38** |  |  |  |  | **1** | **37** |

**Supplementary Table S5** Between-rater agreement of rater A and rater C

| **Likert**  **6-score** | | **rater C** | | | | | | | | | | | | | | | | | |
| --- | --- | --- | --- | --- | --- | --- | --- | --- | --- | --- | --- | --- | --- | --- | --- | --- | --- | --- | --- |
|  |  | **MPH** | | | | | | **LEHRHS-OSEM** | | | | | | **LEHRHS-FBP** | | | | | |
|  |  | **-3** | **-2** | **-1** | **1** | **2** | **3** | **-3** | **-2** | **-1** | **1** | **2** | **3** | **-3** | **-2** | **-1** | **1** | **2** | **3** |
| **rater A** | **-3** | **20** | **4** | **1** |  |  |  | **2** | **7** | **1** |  |  |  | **1** | **3** | **1** |  |  |  |
|  | **-2** | **4** | **1** | **1** |  |  |  | **1** | **9** | **5** | **1** |  |  |  | **10** | **9** |  |  |  |
|  | **-1** |  |  |  |  |  |  |  | **2** | **1** | **1** |  |  |  | **2** | **1** |  |  |  |
|  | **1** |  |  |  |  |  |  |  |  | **1** | **1** |  |  |  | **3** | **1** |  |  |  |
|  | **2** |  |  |  | **1** | **1** | **1** |  |  | **1** |  |  |  |  |  |  | **1** |  | **1** |
|  | **3** |  |  |  |  |  | **37** |  |  |  |  |  | **38** |  |  |  |  |  | **38** |

**Supplementary Table S6** Between-rater agreement of rater B and rater C

| **Likert**  **6-score** | | **rater C** | | | | | | | | | | | | | | | | | |
| --- | --- | --- | --- | --- | --- | --- | --- | --- | --- | --- | --- | --- | --- | --- | --- | --- | --- | --- | --- |
|  |  | **MPH** | | | | | | **LEHRHS-OSEM** | | | | | | **LEHRHS-FBP** | | | | | |
|  |  | **-3** | **-2** | **-1** | **1** | **2** | **3** | **-3** | **-2** | **-1** | **1** | **2** | **3** | **-3** | **-2** | **-1** | **1** | **2** | **3** |
| **rater B** | **-3** | **19** | **4** | **2** |  |  |  | **3** | **6** | **1** |  |  |  |  | **9** | **6** |  |  |  |
|  | **-2** | **5** | **1** |  |  |  |  |  | **6** | **2** | **2** |  |  | **1** | **6** | **4** |  |  |  |
|  | **-1** |  |  |  |  |  |  |  | **6** | **5** |  |  |  |  | **3** | **1** |  |  |  |
|  | **1** |  |  |  | **1** | **1** |  |  |  | **1** | **1** |  |  |  |  | **1** |  |  |  |
|  | **2** |  |  |  |  |  | **4** |  |  |  |  |  |  |  |  |  | **1** |  | **1** |
|  | **3** |  |  |  |  |  | **34** |  |  |  |  |  | **38** |  |  |  |  |  | **38** |

**Supplementary Figures**


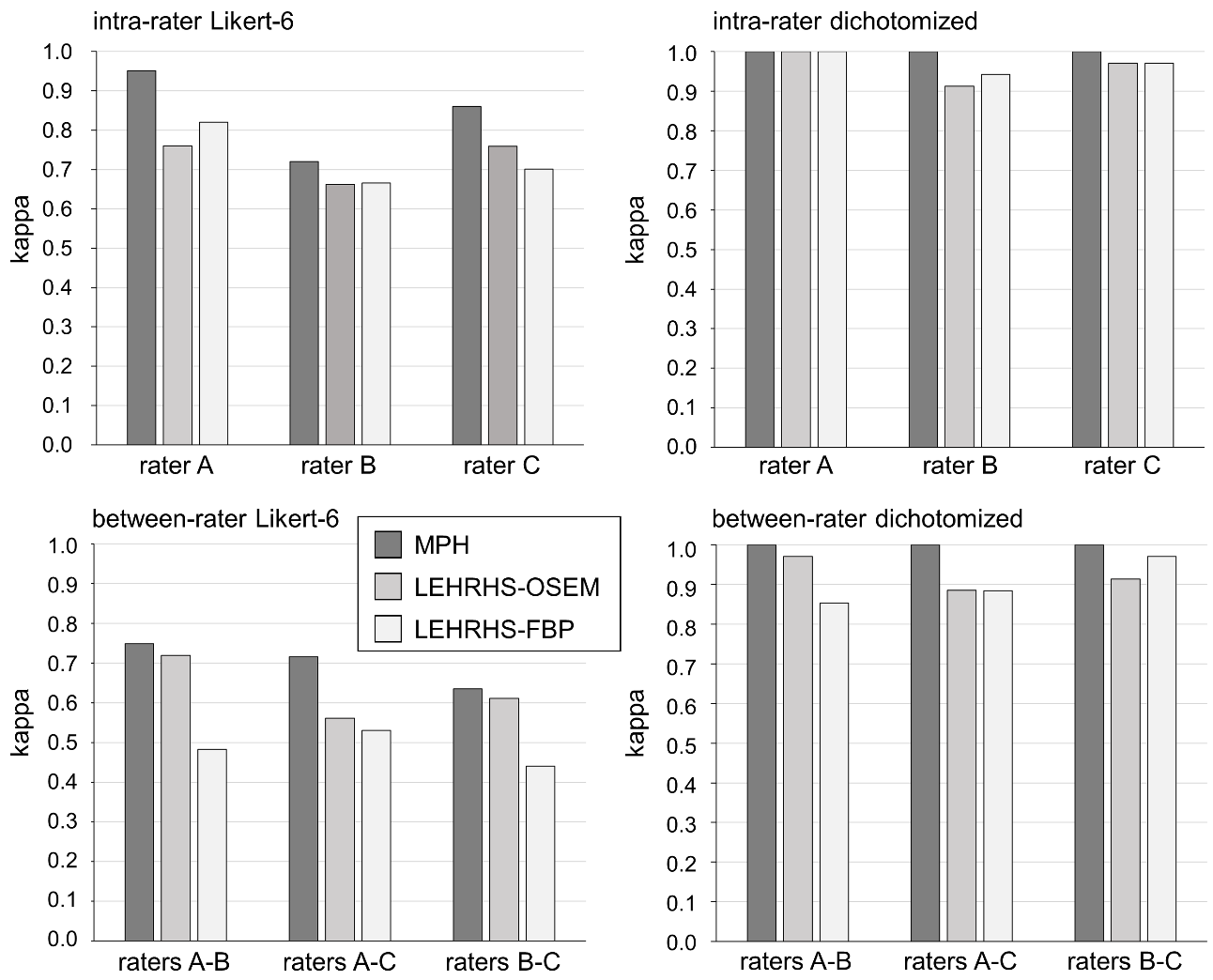


**Supplementary Figure S1** Intra- (top row) and between-rater (bottom row) variability of the visual interpretation of the DAT-SPECT according to the Likert 6-score (left column) and according to the dichotomized Likert 6-score (right column), separately for each individual rater and each pair of raters.


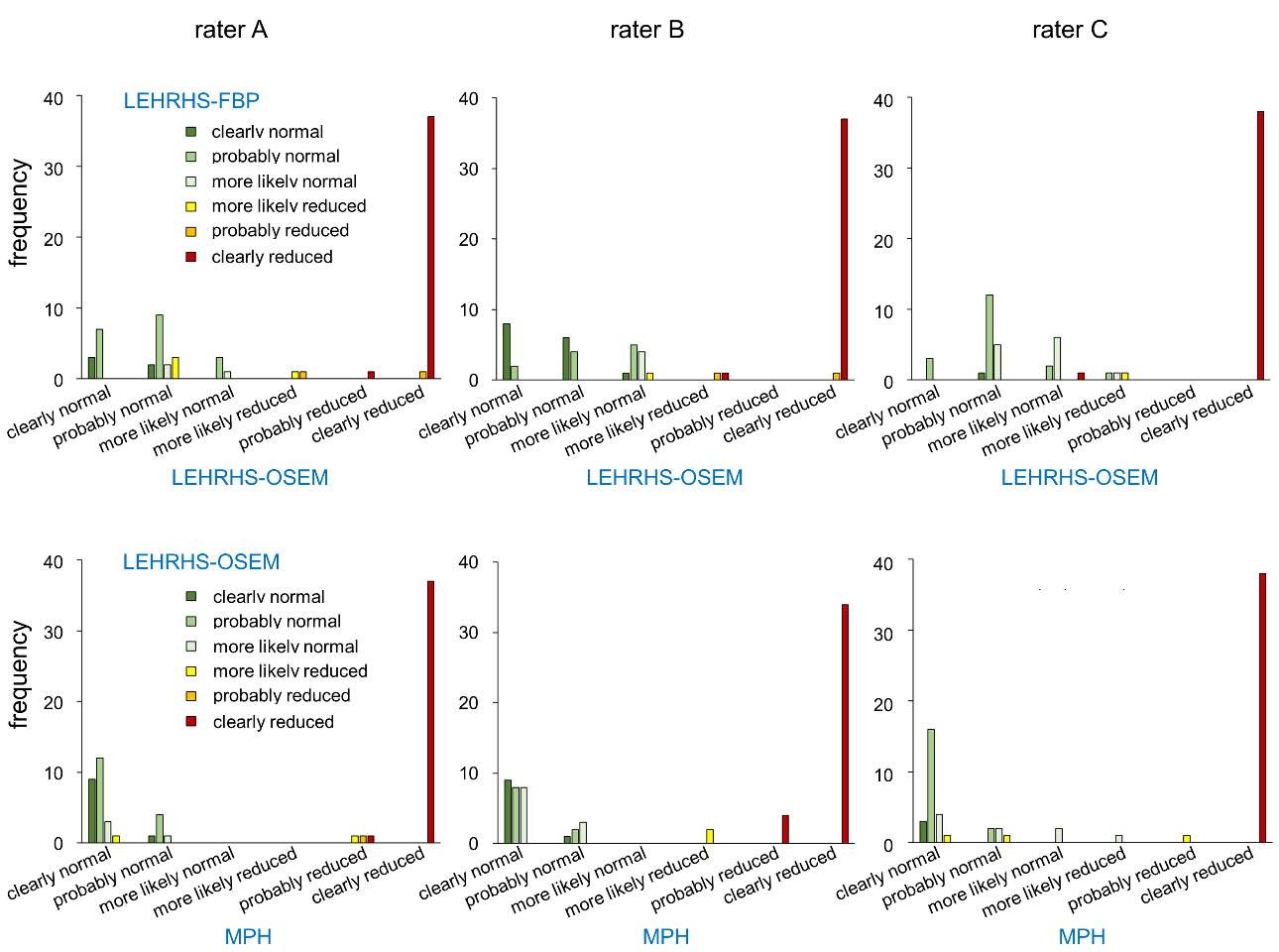


**Supplementary Figure S2** Changes of the intra-rater consensus Likert 6-score between LEHRHS-FBP to LEHRHS-OSEM (top row) and between LEHRHS-OSEM and MPH (bottom row), separately for each individual rater. Changes mainly occurred between the different certainty levels of normal DAT-SPECT, particularly from lower to higher certainty of being normal.
